# Farm-Level Risk Factors for Fish-Borne Zoonotic Trematode Infection in Integrated Small-Scale Fish Farms in Northern Vietnam

**DOI:** 10.1371/journal.pntd.0000742

**Published:** 2010-07-13

**Authors:** Van Thi Phan, Annette Kjær Ersbøll, Khue Viet Nguyen, Henry Madsen, Anders Dalsgaard

**Affiliations:** 1 Department of Large Animal Sciences, Section of Veterinary Epidemiology, Faculty of Life Sciences, University of Copenhagen, Frederiksberg, Denmark; 2 Centre for Environment and Disease Monitoring in Aquaculture, Research Institute for Aquaculture No.1, Tu Son, Vietnam; 3 Centre for Health Research and Development, Department of Veterinary Disease Biology, Faculty of Life Sciences, University of Copenhagen, Frederiksberg, Denmark; 4 Department of Veterinary Disease Biology, Faculty of Life Sciences, University of Copenhagen, Frederiksberg, Denmark; Khon Kaen University, Thailand

## Abstract

**Background:**

Northern Vietnam is an endemic region for fish-borne zoonotic trematodes (FZT), including liver and intestinal flukes. Humans acquire the FZT infection by eating raw or inadequately cooked fish. The production of FZT-free fish in aquaculture is a key component in establishing a sustainable program to prevent and control the FZT transmission to humans. Interventions in aquaculture should be based on knowledge of the main risk factors associated with FZT transmission.

**Methodology/Principal Findings:**

A longitudinal study was carried out from June 2006 to May 2007 in Nam Dinh province, Red River Delta to investigate the development and risk factors of FZT infections in freshwater cultured fish. A total of 3820 fish were sampled six times at two-month intervals from 96 fish farms. Logistic analysis with repeated measurements was used to evaluate potential risk factors based on information collected through questionnaire interviews with 61 fish farm owners. The results showed that the FZT infections significantly increased from first sampling in June to July 2006 (65%) to sixth sampling in April to May, 2007 (76%). The liver fluke, *Clonorchis sinensis* and different zoonotic intestinal flukes including *Haplochis pumilio*, *H. taichui*, *H. yokogawai*, *Centrocestus formosanus* and *Procerovum varium* were found in sampled fish. Duration of fish cultured (sampling times), mebendazole drug self-medication of household members, presence of snails in the pond, and feeding fish with green vegetation collected outside fish farms all had a significant effect on the development of FZT prevalence in the fish.

**Conclusions/Significance:**

The FZT prevalence in fish increased by 11 percentage points during a one-year culture period and the risk factors for the development of infection were identified. Results also highlight that the young fish are already highly infected when stocked into the grow-out systems. This knowledge should be incorporated into control programs of FZT transmission in integrated small-scale aquaculture nursery and grow-out systems in Vietnam.

## Author Summary

Fish are the second intermediate host for fish-borne zoonotic trematodes (FZT). Humans acquire the FZT by eating raw or inadequately cooked fish. Therefore any sustainable program to prevent and control human FZT infection should consider how to minimize the FZT prevalence in fish. Understanding the development in prevalence of FZT in fish and the risk factors involved are of key importance in order to plan and implement an FZT prevention program successfully. During a one-year production cycle in integrated small-scale aquaculture grow-out systems, the FZT prevalence in fish increased by 11 percentage points. Three risk factors associated with the development of the prevalence of FZT infection in fish were identified including presence of snails in the pond, feeding fish with green vegetation collected outside fish farms, and mebendazole medication by the household members. Aquaculture management solutions addressing these three risk factors as well as the high level of FZT infection in juvenile fish stocked in grow-out systems should be found in order to produce fish free of FZT, which are safe for human consumption.

## Introduction

Fish-borne zoonotic trematodes (FZT) belonging to the Opisthorchiidae and Heterophyidae families are important emerging and re-emerging pathogens causing liver and intestinal fluke diseases in human [1], [2]. The life cycle of FZT involves humans and animals such as dog, cat, pig and fish-eating birds as final hosts. The FZT live and develop to adult flukes in the liver or intestines of the final host and produce eggs that are excreted into the environment through faeces. The eggs are ingested by fresh water snails, where they develop to a stage called cercariae. Free swimming cercariae are shed to the water before they penetrate into fish. Inside the fish tissues, cercariae transform into encysted metacercariae [2]–[4]. Humans and animals acquire the FZT infection through consumption of raw, inadequately cooked, dried, salted or pickled fish that harbor infective metacercariae stages [4]–[6]. Keiser and Utzinger [4], [5] estimated that about 681 million people worldwide are at risk of infection and more than 46 million people are infected with liver flukes (*Clonorchis sinensis* and *Opisthorchis* sp.). There are no such estimates available of the number of people infected with intestinal flukes worldwide [4], but the prevalence of infection is believed to be high, e.g. the Red River Delta, Northern Vietnam is endemic for FZT, including both liver and intestinal trematode infections in humans and animals [7]–[9].

The fisheries sector in Vietnam plays an important role in providing food for domestic consumption and generating foreign currencies through export of seafood with aquaculture products contributing significantly to such export. From 1998 to 2008, the aquaculture sector in Vietnam expanded rapidly with the production volume increasing about 5.8 times from 425,000 tons to about 2.5 million tons in 2008. In the same period, the overall export value of seafood increased from 858 million USD to 4,510 million USD [10]. Vietnamese aquaculture systems include marine, brackish water and freshwater aquaculture. The freshwater aquaculture production environments include ponds, ditches, cages, net enclosures and pens in reservoirs, lakes, rivers and channels, and paddy fields. In the Red River Delta of Northern Vietnam, small-scale polyculture farming with several carps species and tilapia is the most important farming system [11]. At the study site in Nam Dinh province, fish are stocked continuously in small-scale grow-out ponds and partial harvest is done to generate extra income or for household consumption. The main stocking season for grow-out ponds is late Spring and early Summer where juveniles are available from the nurseries.

**Figure 1:**
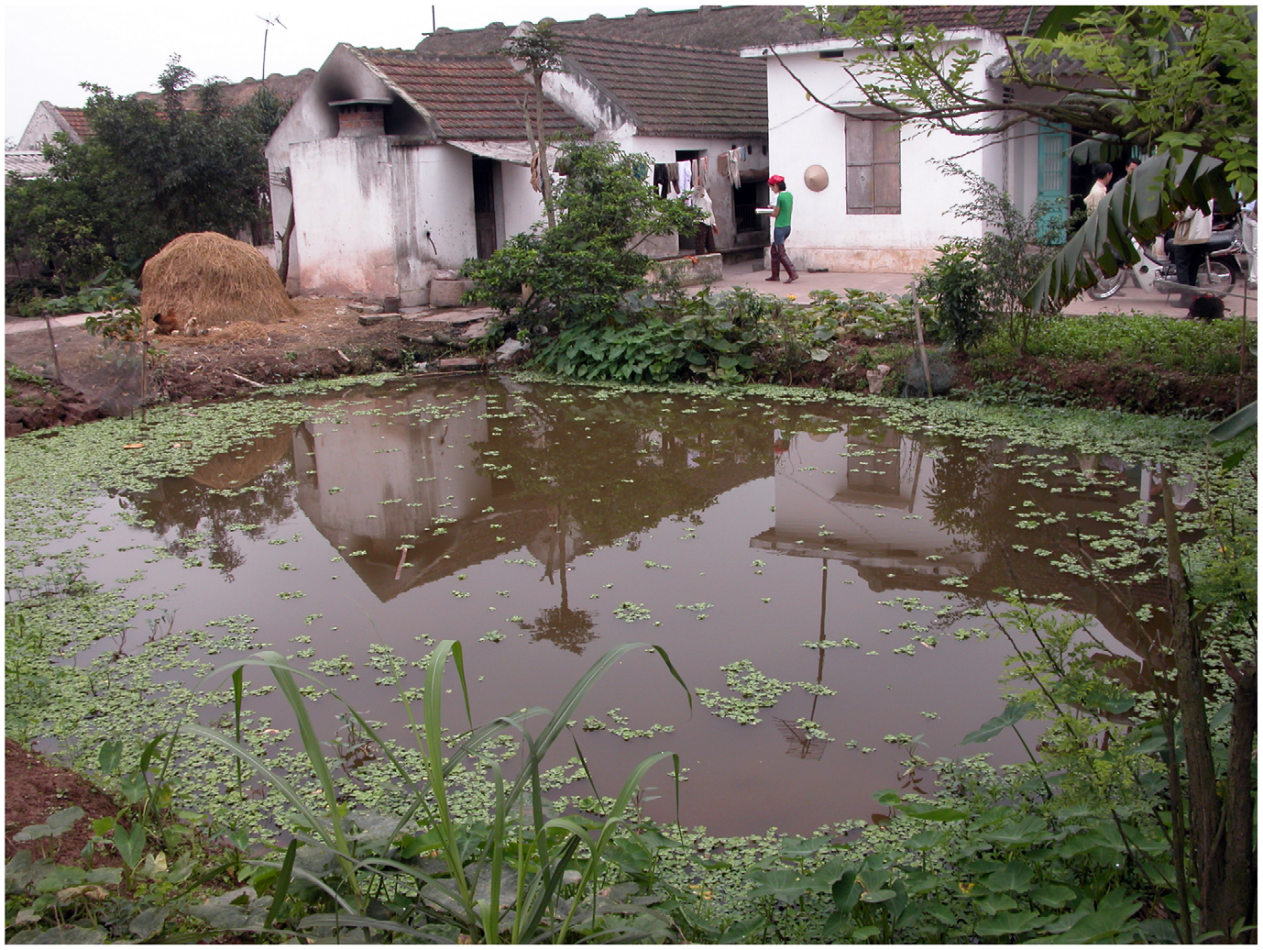
A grow-out pond in Nam Dinh province. Shown a typical grow-out pond in Nam Dinh province. The pond is earthen pond and located near the households.

In Nam Dinh province in particular, is mainly practiced as small-scale family-based systems. The cultured fish are a main protein source for the households and consumed by the families at any time of the year. In Nam Dinh province, as in many other areas in Northern Vietnam, freshwater fish ponds are stocked with multiple fish species and raised in a continuous production cycle. The aquaculture activities are often integrated with livestock, and vegetable production. The grow-out ponds are earthen ponds and often located in backyard of households (Figure 1.). These systems are conducive for the life cycle of FZT as contamination with FZT eggs from animal and human hosts is high and the snail intermediate host are often present in high numbers. There have been a number of educational campaigns to stop people in Nam Dinh province from eating raw or inadequate cooked fish, however, the FZT prevalence in humans in Nam Dinh remains high [8]. It is an arduous task to convince people to stop eating raw and inadequate cooked fish because there is a long tradition for eating such dishes. Therefore, producing fish free of FZT for human consumption is important to prevent such infection in human.

The present study was carried out during a period of 1-year and aimed to investigate the prevalence and the development of FZT infection in fish during a 1-year culture cycle and to assess risk factors for FZT infection in fish.

## Methods

### Ethics statement

The study protocol was approved by the Aquatic animal scientific committee of Research Institute for Aquaculture No.1, Dinh Bang, Tu Son, Bac Ninh, Vietnam. Informed consent was obtained in writing from fish farmers as they submitted a signed request form to participate in the study.

### Study area, design and unit

**Figure 2:**
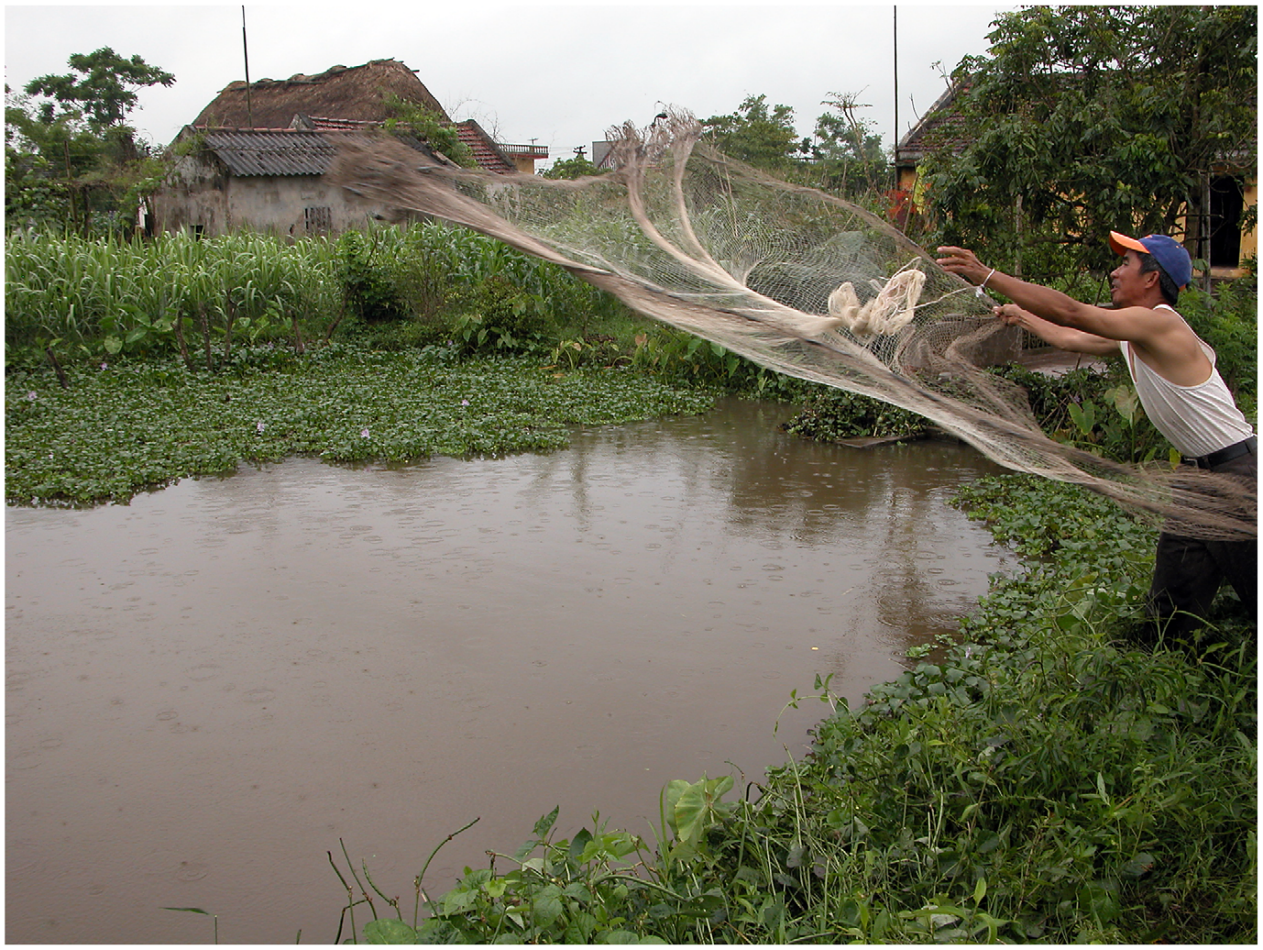
Sampling fish by using cast net. Shown a throw of cast net when sampling fish from a grow-out pond in Nam Dinh.

Nam Dinh province is well- known as an FZT endemic province with high FZT prevalences in humans (64.9%), cats (70.2%) and dogs (56.9%) [8], [9]. The study was conducted in Nghia Lac and Nghia Phu communes in Nam Dinh province, which is located in the Red River Delta in Northern Vietnam (Figure 2). The main stocking season is late Spring and beginning of summer. During other times of the year, juveniles are stocked at convenience by farmers. The stocked juveniles mainly originate from local nurseries. The main species stocked are grass carp (*Ctenopharyngodon idellus*), silver carp (*Hypophthalmichthys molitrix*), common carp (*Cyprinus carpio*), Rohu (*Labeo rohita*), and Mrigal (*Cirrhinus mrigala*). So-called self-recruiting species of fish (SRS fish) are occasionally accidently introduced to the ponds during intake of water from nearby canals. Depending on species, the stocked fingerlings are grown for 8 to 12 months to reach harvest size.

This study was carried out during a 1-year fish production cycle from May 2006 to April 2007 and designed as a longitudinal study with 6 repeated samplings from the same ponds at 2-month intervals. The study unit was the pond at each sampling time.

### Farm selection and fish sampling

Fish farms were selected from a list of households obtained from the local health clinic in each of the two communes. Farms with no fish ponds and farms that participated in a previous human FZT prevalence study reported by Dung *et al.* [8] were excluded because infected individuals were given drug treatment. Farms were randomly selected from the adjusted lists of households. If the farm had more than one pond, then one pond was randomly selected and used for fish sampling throughout the study period. A total of 96 fish farms were investigated.

**Figure 3:**
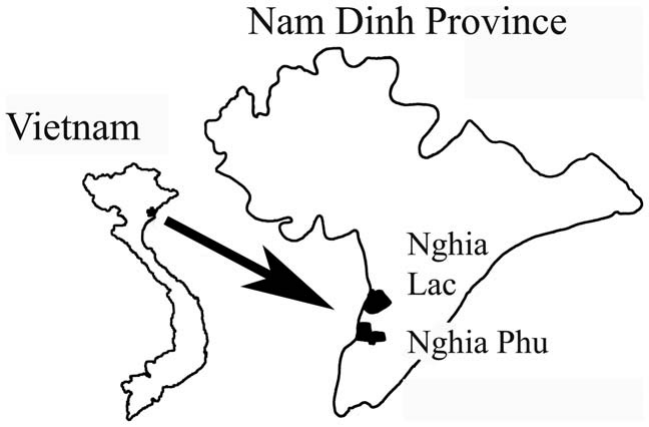
Map of study area. Nam Dinh province is located in the Southern Red River Delta, bordered by Ha Nam province to the North, the Gulf of Tonkin in the South-East, Thai Binh province to the East and Ninh Binh province on the West. Nghia Lac and Nghia Phu communes belong to Nghia Hung district, Nam Dinh province.

Each farm was given one cast net for fish sampling in order to avoid risk of contamination between ponds. In each pond, a cast net was thrown in each of the four corners and in the middle of the pond (Figure 3.). All fish caught from the five throws were put in a bucket and 10 fish were randomly selected (irrespective of fish species). However, if less than 15 fish were caught in a pond then the all fish were selected for analyses.

### Analysis for FZT metacercariae in fish

Fish samples were preserved on ice and transported within 12 hours to the parasitological laboratory of the Centre for Environment and Disease Monitoring in Aquaculture (CEDMA), Research Institute for Aquaculture No.1 (RIA1), Dinh Bang, Tu Son, Vietnam. In the laboratory, fish samples were kept in a refrigerator at 4°C for no more than five days before being processed. The length and weight of each fish were recorded before being processed and digested in 1% pepsin for the release of metacercariae following procedures previously described [12] and modified as described by Chi *et al.* [13]. Small fish (less than 200 g in weight) were ground and digested whole. For larger fish (>200 g), the whole fish was ground, mixed well and a 50 g sub-sample was obtained and digested for recovery of metacercariae.

Metacercariae identification skills among staff were evaluated at the beginning of the study to ensure that FZT species were correctly identified by providing test samples for each staff to identify. Three times during the study period, the identification of the metacercariae was verified by experimental infection of mice and subsequent identification of adult flukes recovered. The experimental infections and identification of adult flukes followed previously described procedures and criteria [14]. The identity of metacercariae verified in animal experiments was subsequently confirmed to be correctly identified.

### Snail sampling and cercariae analysis

Data on snails were extracted from Dung *et al.* [15]. Snail sampling was conducted by the same person in all ponds during the morning hours for 30 minutes per pond using a scooping method and/or hand-picking from the pond. Snails were transferred to plastic containers and transported alive to the laboratory where they were identified according to keys by Brandt [16], Thanh [17] and Frandsen and Christensen [18]. Snails were then examined for trematode infections using one or more of three methods depending on snail size, i.e. shedding, crushing and cutting, and cercariae were identified only to major group [15].

### Questionnaire development and interviewing

A questionnaire including 33 questions was formulated based on information obtained during group discussions with international experts, scientists at CEDMA and fishery staff at provincial level about potential risk factors for FZP infection at farm level. Information was obtained about farm practices in relation to the pond management, animal reservoir host and human waste such as how often and how farmers clean the pond, water source, practices for keeping animals such as dogs, cats, pigs, chicken and ducks, toilet facilities and others. The draft questionnaire was pretested by interviewing aquaculture research staff at CEDMA and five farmers in each commune with the farmers subsequently being excluded from the study. Needed adjustments were then made to the questionnaire which also included a section for recording observations made during the farm visit.

Four persons were involved in interviewing the managers of the farms. Before the interviews were initiated, the four persons discussed and agreed upon the method of interviewing and filling out the questionnaires. Each farm was interviewed one time in the connection with the first sampling by two persons. One person was always the interviewer and the second person was observing the interview and took notes about observed farm management practices, presence of animal and toilet facilities etc. in order to validate the answers given by the manager of the farm.

Most of the questions in the questionnaire were closed with 2 to 4 possible answers. Open questions were used to obtain information about pond area, number of fish stocked and yield. The questionnaire can be obtained from corresponding author.

### Derived questionnaire variables

All questionnaire variables were initially evaluated using frequency distributions in order to obtain variables for further analysis. For some questions, most farmers gave the same answers. Hence these variables (questions) were excluded from the analysis because they did not contribute to explaining differences in transmission due to skewed distribution. Furthermore, many of the questions were not independent. Therefore, new variables were derived based on biological similarity and normal daily practices. As an example, a variable “animal fed with live fish” was derived based on specific information about how pigs, dogs and cats were fed or seen eating live fish. Area of pond was categorized into less than 300 m^2^ and ponds equal to or more than 300 m^2^. The source of green vegetation for feeding grass carp was originally one question in the questionnaire. However, two new variables were derived as feeding grass carp with additional vegetation originating from inside the garden (yes/no) and feeding grass carp with additional green vegetation originating from outside the garden (yes/no). The term of “additional green vegetation” here includes some types of grasses (sometimes from flooded areas) or aquatic plants collected from other habitats. The remaining variables were derived by aggregating some of the answers possibilities.

### Statistical analysis

Data were analysed using the Statistical Analysis System (SAS®, version 9.1). The dependent variable was number of FZT infected fish out of number of examined fish in the pond at each sampling time. Descriptive analysis was performed by means of number of farms, number of infected fish, number of sampled fish, overall FZT prevalence by sampling times and potential risk factors for FZT transmission at the farms. Initially, two-by-two tables were used to evaluate confounding between potential farm-level risk factors.

The effect of potential risk factors on the prevalence of FZT infection in fish was evaluated using a logistic analysis with repeated measurements. The potential risk factors were included in the analysis. Farm nested within commune was included as a random effect. The autocorrelation between repeated measurements was taken into account by introducing a 1^st^ order autoregressive correlation structure in the model. Backward elimination was applied to obtain the main effect resulting model. Interactions (2-way) between variables in the main effect resulting model were evaluated. In order to evaluate confounding between excluded potential risk factors with risk factors in the resulting model, each excluded variable was then added to the resulting model one by one for testing significance. Odds ratios were calculated only for significant effects. A *P*-value <0.05 was considered significant.

## Results

### Descriptive analysis

Initially, there were 96 farms included in the study. However, during the 1-year study period some farms terminated fish culture with only 61 farms being sampled five times while 52 farms were sampled six times. A total of 3820 fish originating from 61 farms were examined for FZT.

#### FZT in fish and snail species

Among the fish species sampled for FZT examination, Rohu, Silver carp, Mrigal and Grass carp were the dominating species comprising 85% of the total number of fish analyzed. Table 1 lists the different fish species, the percentage infected with FZT, and the parasite species found. The weight of fish sampled ranged from 8 to 1400 g depending on fish species. The liver fluke, *Clonorchis sinensis*, was only found in 1 of 1185 Silver carps analyzed. Different intestinal flukes including *Haplochis pumilio*, *H. taichui*, *H. yokogawai*, *Centrocestus formosanus* and *Procerovum varium* were commonly found in most fish species. *Haplochis pumilio* was recovered from all 18 fish species and the main cultured fish species (Grass carp, Rohu, Mrigal, Silver carp and Pacu) were infected by five different zoonotic intestinal trematodes. Non-zoonotic Exorchis spp. were found as metacercariae in fish in this study area (unpublished data).

**Table 1:** FZT diversity and prevalence in fish from small-scale farms in Nam Dinh, Vietnam.

				FZT species found^c^					
Fish species (*Latin name*)	N_f_ (%)^a^	N_f_ (%)^a^	N_FZT_/N (%)^b^	*Cs*	*Ht*	*Hp*	*Hy*	*Pr*	*Ce*
Rohu (*Labeo rohita*)	1290(33.75)	1290(33.8)	745 (58)	−	+	+	+	+	+
Silver carp (*Hypophthalmichthys molitrix*)	1185(31.0)	1185(31)	1024 (86)	+	+	+	+	+	+
Mrigal (*Cirrhinus mrigala*)	424(11.09)	424(11.1)	313 (74)	−	+	+	+	+	+
Grass carp (*Ctenopharyngodon idellus*)	351(9.18)	351(9.2)	305 (87)	−	+	+	+	+	+
Crucian carp (*Carrasius auratus*)	196(5.13)	196(5.1)	127 (65)	−	−	+	+	−	−
Barbel chub (*Squaliobarbus curriculus*)	121(3.17)	121(3.2)	86 (71)	−	+	+	−	+	+
Pacu (*Piaractus brachypomum*)	75(1.96)	75(2.0)	55 (73)	−	+	+	+	+	+
Tilapia (*Oreochromis niloticus*)	54(1.41)	54(1.4)	13 (24)	−	−	+	−	−	−
Common carp (*Cyprinus carpio*)	44(1.15)	44(1.2)	36 (82)	−	−	+	+	−	+
Mud carp (*Cirrhinus molitorella)*	22(0.57)	22(0.6)	8 (38)	−	−	+	−	−	−
Catfish (*Clarias batrachus*)	9(0.24)	9(0.2)	7 (78)	−	−	+	+	+	+
Snake-head fish (*Channa orientalis*)	8(0.21)	8(0.2)	7 (88)	−	−	+	−	−	+
Climbing perch (*Anabas testudineus*)	31(0.81)	31(0.8)	29 (94)	−	−	+	−	+	+
Silver barb (*Barbonymus gonionotus*)	6(0.16)	6(0.2)	3 (50)	−	−	+	−	+	−
Sharpbelly (*Hemiculter leucisculus*)	3(0.08)	3(0.1)	1 (33)	−	−	+	−	−	−
Big head carp (*Hypophthalmichthys nobilis*)	1(0.03)	1(0.03)	1 (100)	−	−	+	−	−	−
Bronze featherback (*Notopterus notopterus*)	1(0.03)	1(0.03)	1 (100)	−	−	+	−	−	−
Unidentified	1(0.03)	1(0.03)	1 (100)	−	−	+	−	+	−
TOTAL	3822(100)	3822(100)	2762 (72)						

a Nf: Number of specific fish species, %: percentage of each fish species.

b N_FZT_ is number of FZT infected fish; N is number of fish,%: prevalence of FZT in each fish species.

c *Cs* is *Clonorchis sinensis*; *Ht* is *Haplochis taichui*; *Hp* is *H. pumilio*; *Hy* is *H. yokogawai*; *Pr* is *Procerovum varium*; *Ce* is *Centrocestus formosanus*.

The snail fauna in ponds was dominated by species of the Viviparidae family (53% of all snails collected) and Thiaridae family (41%), but species of the Ampullaridae family (5.5%) and Bithynidae (0.4%) were also present. *Stenothyra messageri* and different pulmonate snail species were occasionally found. Among Viviparidae, only *Angulyagra polyzonata* was found while within the Thiaridae, *Melanoides tuberculata* was dominating (75%); the other species in this family were *Thiara scabra* (22%), *Tarebia granifera* (1.5%) and *Sermyla requetii* (1.7%). The cercariae types recorded from the ponds were echinostomes, gymnocephalous, monostome, parapleurolophocercous (no pleurolophocercous cercariae were found in ponds), and xiphidio cercariae. The trematode species found in the fishes all belonged to the Heterophyidae and these produce cercariae of the parapleurolophocercous (or pleurolophocercous) group, which was the most common type found in the ponds (37% of all infections found). In the ponds, these cercariae were found exclusively in species of the Thiaridae.

#### FZT development in the 1-year study period

The FZT prevalence in fish sampled increased 11 percentage points over the period, from 65% in the initial sample to 76% in the final sample (Table 2). The increase in FZT prevalence in Nghia Lac and Nghia Phu communes were 9 and 12 percentage points, respectively.

**Table 2:** Descriptive analysis of potential risk factors for FZT infection in fish from small-scale farms.

			*Sampling 1 (Jun–Jul)	Sampling 2 (Aug–Sep)	Sampling 3 (Oct–Nov)	Sampling 4 (Dec–Jan)	Sampling 5 (Feb–Mar)	Sampling 6 (Apr–May)	
Potential risk factor	Level	N_hh_	N_FZT_/N_f_ (%)	N_FZT_/N_f_ (%)	N_FZT_/N_f_ (%)	N_FZT_/N_f_ (%)	N_FZT_/N_f_ (%)	N_hh_	N_FZT_/N_f_ (%)
***Overall***		61	408/624 (65)	395/577 (68)	428/568 (75)	429/580 (74)	423/563 (75)	52	357/473 (76)
Commune	Nghia Lac	34	244/368 (66)	224/334 (67)	231/317 (73)	212/311 (68)	229/317 (72)	31	215/286 (75)
	Nghia Phu	27	164/256 (64)	171/243 (70)	197/251 (79)	217/269 (81)	194/246 (79)	21	142/187 (76)
***Fish pond management***									
Area of pond	≤300 m^2^	30	218/314 (69)	192/271 (71)	196/269 (73)	219/292 (75)	217/284 (76)	24	160/215 (74)
	>300 m^2^	31	190/310 (61)	203/306 (66)	232/299 (76)	210/288 (73)	206/279 (74)	28	197/258 (76)
Source of water used in pond	Canal	47	311/488 (64)	302/457 (66)	338/450 (75)	356/475 (75)	338/446 (76)	39	273/356 (77)
	Rain	14	97/136 (71)	93/120 (76)	90/118 (76)	73/105 (70)	85/117 (73)	13	84/117 (72)
Frequency of pond preparation	Yearly	22	156/243 (64)	143/208 (69)	142/197 (72)	166/217 (77)	152/190 (80)	21	133/179 (74)
	>1 year	36	224/343 (65)	223/334 (67)	247/331 (75)	237/328 (72)	246/331 (74)	29	199/267 (75)
Use lime in pond	Yes	32	227/333 (68)	213/301 (71)	223/302 (74)	211/293 (72)	227/288 (79)	27	199/264 (75)
	No	26	153/253(61)	153/241 (64)	166/226 (75)	192/252 (76)	170/233 (73)	23	133/182 (73)
Source of fish fry	Local	29	204/316 (65)	204/289 (71)	240/309 (78)	224/309 (73)	214/281 (76)	25	195/248 (79)
	Don't know	23	129/209 (62)	142/212 (67)	135/182 (74)	158/203 (78)	152/209 (73)	18	99/132 (75)
Origin of additional green vegetation**	Outside farm	51	344/519 (66)	336/483 (70)	365/469 (78)	361/480 (75)	355/468 (76)	43	308/396 (78)
	Inside farm	10	64/105 (61)	59/94 (63)	63/99 (64)	68/100 (68)	68/95 (72)	9	49/77 (64)
Presence of snail**	No	42	275/422 (65)	254/385 (66)	253/363 (70)	277/384 (72)	282/381 (74)	36	219/309 (71)
	Yes	19	133/202 (66)	141/192 (73)	175/205 (85)	152/196 (78)	141/182 (78)	16	138/164 (84)
***Animal reservoir host***									
Rear pig	No	13	84/121 (69)	91/123 (74)	92/113 (81)	103/131 (79)	80/105 (76)	11	72/96 (75)
	Yes	48	324/503 (64)	304/454 (67)	336/455 (74)	326/449 (73)	343/458 (75)	41	285/377 (76)
Have cat	No	15	103/169 (61)	98/139 (71)	98/140 (70)	92/131 (70)	94/126 (75)	13	75/113 (66)
	Yes	46	305/455 (67)	297/438 (68)	330/428 (77)	337/449 (75)	329/437 (75)	39	282/360 (78)
Have dog	No	9	53/83 (64)	68/94 (72)	65/82 (79)	75/90 (83)	58/71 (82)	9	62/76 (82)
	Yes	52	355/541 (66)	327/483 (68)	363/486 (75)	354/490 (72)	365/492 (74)	43	295/397 (74)
Rear duck	No	47	315/471 (67)	316/469 (67)	330/431 (77)	332/440 (76)	320/435 (74)	38	250/332 (75)
	Yes	14	93/153 (61)	79/108 (73)	98/137 (72)	97/140 (69)	103/128 (81)	14	107/141 (76)
Rear chicken	No	21	154/235 (66)	155/226 (69)	184/232 (79)	174/232 (75)	161/221 (73)	20	123/170 (72)
	Yes	40	254/389 (65)	240/351 (68)	244/336 (73)	255/348 (73)	262/342 (77)	32	234/303 (77)
Use animal manure	Pond, garden	16	116/170 (68)	114/160 (71)	127/157 (81)	148/186 (80)	108/147 (74)	13	87/112 (78)
	Others	43	281/433 (65)	267/396 (67)	283/389 (73)	267/375 (71)	302/398 (76)	37	256/339 (76)
Seen animal eat live fish	No	41	274/414 (66)	269/377 (71)	285/377 (76)	267/365 (73)	280/377 (74)	34	230/302 (76)
	Yes	20	134/210 (64)	126/200 (63)	143/191 (75)	162/215 (75)	143/186 (77)	18	127/171 (74)
Feeding animal fish waste	No	30	209/313 (67)	206/291 (71)	229/300 (76)	224/299 (75)	207/284 (73)	27	189/244 (78)
	Yes	31	199/311 (64)	189/286 (66)	199/268 (74)	205/281 (73)	216/279 (77)	25	168/229 (73)
***Human waste and other***									
Type of toilet	Closed	38	240/383 (63)	251/259 (70)	269/356 (76)	282/377 (75)	276/368 (75)	31	211/264 (80)
	Open	23	168/241 (70)	144/218 (66)	159/212 (75)	147/203 (72)	147/195 (75)	21	146/209 (70)
Discharging waste from toilet	Canal	4	13/27 (48)	26/32 (81)	23/30 (77)	32/42 (76)	23/31 (74)	3	19/23 (83)
	Pond	13	99/146 (68)	98/137 (72)	130/152 (86)	119/146 (82)	108/138 (78)	12	100/122 (82)
	Garden	20	126/205 (62)	129/185 (70)	114/164 (70)	133/183 (73)	132/185 (71)	15	89/117 (76)
Heard about intestinal fluke	No	43	293/435 (67)	288/394 (73)	321/406 (79)	317/404 (75)	310/411 (75)	34	245/319 (77)
	Yes	18	115/189 (61)	107/183 (59)	107/162 (66)	112/156 (72)	113/152 (74)	18	112/154 (73)
Mebendazole drug self-medication**	No	37	237/385 (62)	243/372 (65)	270/358 (75)	260/372 (70)	243/345 (70)	30	215/290 (74)
	Yes	24	171/239 (72)	152/205 (74)	158/210 (75)	169/208 (81)	180/218 (83)	22	142/183 (78)

*N_hh_: Number of households; N_f_: number of fish sample; N_FZT_: number of fish infected with FZT; %: prevalence of infected fish.

**Identified risk factors.

Table 2 shows the descriptive analysis of potential risk factors for FZT infection in fish during the 1-year study period. The FZT prevalence increased by 12 percentage point increase in fish fed additional vegetation collected outside the farm garden, but only 3 percentage points when fed with green vegetation collected within the farm garden. Fish cultured in ponds with snails had an 18 percentage points increase in FZT prevalence, while fish from ponds without snails only showed a 6 percentage points increase in FZT prevalence. In farm households that kept cat(s), the FZT prevalence in fish increased by 10 percentage points, while in farms that did not keep cats the increase was 6 percentage points. If household members had taken a standard round worm treatment with mebendazole during the previous year, the FZT prevalence in fish increased by 13 percentage points, while in those that had not taken treatment showed a 6 percentage points increase in FZT prevalence.

### Risk factors for FZP infection in freshwater fish cultured in small-scale integrated ponds

Table 3 presents the final model of risk factors for FZT infection in fish at farm level. Duration of culture period (sampling time) had a significant effect on the development of FZT infected fish (*P* = 0.012). The odds of FZT prevalence in fish at sampling time 3, 5 and 6 was more than 1.6 times higher than at sampling time 1. Farms with household members that took self-medication with antihelminthic worm tablets had 1.52 time higher risk of FZT infection than farms with household members that did not take such treatment (*P* = 0.015).

**Table 3:** Parameter estimate of farm-level risk factors for FZT infection in fish from small-scale farms.

Variable	Level	Estimate*	SE	OR	95% CI	*P* value
Intercept		−0.013	0.237			
Sampling times						0.012
	1 (Jun–Jul)	0^a^	-	1.00	-	
	2 (Aug–Sep)	0.141^ab^	0.150	1.15	0.87–1.53	
	3 (Oct–Nov)	0.503^c^	0.166	1.65	1.19–2.29	
	4 (Dec–Jan)	0.401^bc^	0.165	1.49	1.08–2.06	
	5 (Feb–Mar)	0.496^c^	0.169	1.64	1.18–2.28	
	6 (Apr–May)	0.480^bc^	0.178	1.62	1.14–2.89	
Snails present in pond						0.022
	Yes	0.419	0.177	1.52	1.07–2.15	
	No	0	-	1.00	-	
Origin of additional green vegetation fed to grass carp						0.034
	Inside farm	0	-	1.00	1.05–2.41	0.034
	Outside farm	0.462	0.213	1.59	-	
Mebendazole drug self-medication						0.015
	Yes	0.424	0.169	1.53	1.1–2.13	0.015
	No	0	-	1.00	-	
Autocorrelation		0.195	0.076			

*Parameter estimate with the same superscript letter are not significant at a 5% significant level.

SE is standard error; OR is odds ratio; 95% CI is 95% confidence interval for OR; *P* is overall P-value.

Odds of FZT infection in fish from ponds with snails was 1.52 times higher (*P* = 0.022) than if no snail was seen present. Farms feeding green vegetation supplied from outside their garden had significantly higher FZT infection (OR = 1.59) compared to fish from farms that fed green vegetation originating from their own garden.

## Discussion

The data we present here form the first study of risk factors for FZT development in fish based on a longitudinal study design. Prevalence of FZT in fish was high in these grow-out ponds and we identified a number of factors that promote transmission of these parasites to fish.

The FZT prevalence was found to be high at the beginning of main stocking season of fingerlings with a subsequent increase in FZT prevalence in grow-out ponds increased from 65% to 76% during the 1-year culture period. The juveniles fish obtained from the local nurseries were highly infected when stocked in the grow-out ponds studied (Van Thi Phan, personal communication). Therefore, it is important that nurseries are a focal point for interventions to prevent juvenile fish from becoming infected with FZT. The larger fish in grow-out ponds seem to be more resistant to FZT infection as there was only 11 percentage points increase in infection during the 1-year culture period. Minimizing the FZT transmission in both juveniles and ready-to-harvest fish is important in preventing FZT transmission to humans since it is normally recognized very difficult to change people's eating habits and stop them from eating raw fish, in particular when such habits are rooted in old cultural traditions.It was also found that the increase in FZT prevalence was faster (3 to 7%) during the initial culture period from June–November and subsequently remained relatively lower and stable (1.2 to1.4% through the remaining period at sampling intervals 4, 5 and 6). Sampling time 4 was at the end of lunar year where the farm household members begin harvesting fish to be consumed during the New Year celebration, but also the time where new fingerlings are stocked into the ponds. At this time of the year, the water temperatures in Northern Vietnam usually range between 14–20°C compared, to the summer period (May to October), when temperatures normally range from 28–35°C. It has previously been shown that snails stop shedding cercariae into water at low water temperatures [19]. Further studies are needed to assess the impact of changing water temperatures on presence of snails and their shedding of cercariae.

We only found *C. sinensis* (liver fluke) in a single fish which was surprising since Dung *et al.* [8] based on faecal (stool) examinations as well as identification of collected adult flukes reported high prevalence of *C. sinensis* in people from the same study area in Nam Dinh province. This could mean that people acquire liver flukes infections from eating raw or inadequately cooked fish from other sources, e.g. from natural habitats or other places than Nam Dinh province. Intestinal fluke species recovered from this study were similar with those reported in Nghe An province [13] and elsewhere in Southern provinces [20], [21], except that *Stellantchasmus falcatus* were not found in our study.

Snails are the first intermediate host for FZT and its presence is essential for further transmission and infection of fish. Snail species belonging to Thiaridae family are a common first intermediate host for intestinal FZT [19], [22] while species of the bithynidae are hosts for *C. sinensis* [15]. In the present study, species of snails of the Thiaridae family, important vectors of FZT, were one of the dominating snail types found in ponds. According to Schell (1985) only pleurolophocercous cercariae are produced within the families Opistorchidae and Cryptogonimidae, while both parapleurolophocercous and pleurolophocercous cercariae are found within the Heterophyidae (intestinal trematodes). In other habitats such as canals and rice fields associated with the fish ponds in Nam Dinh, species of the Bithynidae and Stenothyridae are abundant, but also in these habitats parapleurolophocercous cercariae is the most common type found in snails [15]. Dung *et al.* [15] found that parapleurolophocercous constituted 40.3% of all infections found in snails, while pleurolophocercous only constituted 0.3%, but these could thus potentially belong to three different families. One species belonging to the Cryptogonimidae, Exorchis spp. (not zoonotic), has been found as metacercariae in fish in this area (unpublished). Identification by PCR of cercariae similar to the most common type of parapleurolophocercous cercariae found in our study showed it to be *H. pumilio* [23]. Clearly, there is a need for more detailed identification of cercariae using molecular techniques to clarify host parasite relationships in the study area and elsewhere.

It is a common practise of farmers to feed additional green vegetation to grass carps that are cultured together with other fish species in the same pond. Collection of green vegetation from external sources can introduce infected snails attached to such vegetation. FZT eggs originating from nearby wild animals and humans may also be introduced as such eggs could be present in the mud attached to roots of floating plants. In any case, the practice of adding vegetation to ponds was associated with a significant increased risk of FZT infection in fish. Water intake to the ponds is also a potential risk factor for introducing snails and cercariae into the ponds, but it was not found significantly associated with FZT infection in this study. This may be because the farmers studied use primarily rain water for their ponds, although water from nearby canals may be introduced into the ponds during flooding in rainy season. Thus, further studies are needed to assess the relative importance of introducing snails and cercariae into the ponds through contaminated green fodder and surface run-off into the ponds and through different water sources used to fill the ponds. Similarly, the relative impact of interventions to control such potential risk factors in FZT transmission, e.g. feeding only pelleted feed, preventing surface run-off into ponds, lining of pond embankments to control vegetation and snail populations needs to be assessed. The management of fish feed has previously been proposed as one intervention to control the introduction of snails [24].

The common standard treatment among people in the study area against infection with soil-transmitted round worms is the anthelmintic mebendazole. However, this drug does not have an effect on FZT. Fish cultured in household ponds where the members took this drug had a significantly higher risk of FZT infection compared to fish at farms, where the household members did not take such treatment.. The higher risk of FZT infection in fish associated with mebendazole treatment of household members is difficult to explain. This risk may occur simply by chance. However, one of the most common side effects of mebendazole is diarrhea [25]. Diarrhea would lead to more frequent defecation and this combined with more loose stools and a common open defaecation practice in the study area could increase the risk of faecal (egg) contamination of ponds and other habitats. It may also be speculated that although adult intestinal flukes may be inactivated at some level by mebendazole, eggs may still be viable and excreted at higher rates [26]. Further investigations on this are needed.

Even though the presence of cats on the farm did not have a significant effect on FZT prevelence in fish in this study, it should still be considered to include control measures for cats as the farms that kept cats showed twice as high increase in FZT prevalence in fish compared to farms without cats. Cats have been shown as an important final host for sustaining the life cycle of FZT [9], but their importance in FZT transmission may be questioned as cats tend to deposit faeces away from the pond [26].

Cast net were used in the study to ensure that the fish were randomly sampled and this technique is considered as a good technique for random sampling of fish in aquaculture [27]. However, aquatic plants were often present in varying quantities in the small-scale farm ponds studied. Therefore in some cases, selection bias may have occurred as the fish could escape when the net could not reach the bottom of the ponds due to the presence of aquatic plants.

From the final model of risk factors, it is suggested that snail control either by better pond preparation practices, biological control measures (e.g. stocking of snail eating fish), and ensuring that green vegetation fodder is collected from FZT free areas are important interventions to reduce FZT transmission. Further, better general human hygenic behaviour should be promoted, in particular when individuals take mebendazole drugs, to avoid shedding FZT eggs into the pond environment. However, any practical control strategy for FZT infection in fish farms should be pre-tested and evaluated before being introduced to farmers to apply at their farms.

The results from the present study provide together with findings from other studies in Vietnam on risk factors for FZT infection in humans and animals [7]–[9] important knowledge about FZT transmission that can be used to establish sustainable measures to reduce and control FZT infection in fish raised in grow-out ponds. As juveniles fish already showed high FZT prevalence, any interventions should focus on controlling FZT transmission in nurseries as they appear to be hot spots for FZT infection.
